# A systematic review protocol of stigma among children and adolescents with epilepsy

**DOI:** 10.1186/s13643-019-0940-9

**Published:** 2019-01-12

**Authors:** Joseph Kirabira, Jimmy Ben Forry, Alison Annet Kinengyere, Wilson Adriko, Abdallah Amir, Godfrey Z. Rukundo, Dickens Akena

**Affiliations:** 1 Mbarara University of Science and Technology (MUST), P.O. Box 1410, Mbarara, Uganda; 2 Makerere University, Kampala, Uganda

**Keywords:** Stigma, Epilepsy, Children, Adolescents, Prevalence

## Abstract

**Background:**

Epilepsy is a neurological condition that is highly prevalent among children and adolescents with 80% of the victims living in low- and middle-income countries (LMIC). Epilepsy is associated with high levels of both perceived and enacted stigma, which vary geographically and greatly affects the victims’ quality of life and self-esteem. High rates of stigma are also a significant barrier to accessing medical care. Perceived and enacted epilepsy-related stigma is associated with various sociodemographic and clinical factors, which vary from place to place. Therefore, this review will determine the prevalence of stigma of epilepsy among children and adolescents and the associated factors worldwide.

**Methods:**

We will search for literature in PubMed, EMBASE, PsycINFO, and CINAHL databases as well as grey literature. We will also search via Google Scholar to capture relevant literature that may not be in the searched databases. We will then screen reference lists of included studies for more studies. Studies that have documented the prevalence of epilepsy-related perceived or enacted stigma and the associated factors will be eligible for inclusion. Data will be extracted in duplicates using a pre-piloted tool consisting of study and participant characteristics as well as pre-determined factors associated with epilepsy. Heterogeneity will be assessed by a forest plot and quantified by *I*^2^ statistic, and in case it is high, results will be reported as a narrative and it will further be explored by subgroup analysis. In case of homogeneity, meta-analysis will be done. Bias will be assessed using a critical appraisal tool developed for prevalence studies. The strength of evidence among the studies will be assessed using the GRADE approach.

**Discussion:**

Findings from this review will document the burden of stigma of epilepsy and the common contributing factors, which will form the building blocks of interventions that address this health challenge.

**Systematic review registration:**

PROSPERO CRD42017058957

## Background

Epilepsy is a brain disorder characterized by at least two unprovoked seizures more than 24 h apart or one unprovoked seizure when the risk for another is known to be high (> 60%): reflex seizures and seizures that are part of an epilepsy syndrome [1]. It has a predominant childhood onset, hence being highly prevalent among children and adolescents [2–5]. Unlike other neurological disorders, epilepsy is associated with high levels of stigma, which stretches as far back as the 1800s when people with epilepsy used to be kept in prisons, lunatic asylums, leprosy colonies, and special institutions because people considered it a disgracing illness [6]. The epilepsy-related stigma can take various forms; however, enacted and perceived stigmas are more debilitating [7–9]. Perceived stigma focuses on the individual’s experience of being unfairly treated because of having an undesirable and deeply discrediting attribute [10] (in this case epilepsy) whereas enacted stigma deals with the expressed attitudes of others towards the individual with epilepsy [11]. The presence of stigma among children and adolescents has been associated with low self-esteem, poor quality of life, retardation in neurocognitive development, poor academic progress [12–14], poor health-seeking behaviors, and increased rates of mortality (mainly through suicide) [15, 16].

A study conducted among 15 European countries found the prevalence of epilepsy-related stigma in high-income countries (HIC) to be 51% with 18% of participants reporting high stigmatization [17]. However, several studies in low- and middle-income countries (LMIC) have found epilepsy perceived stigma level ranging from 31 to 69% [18–21]. The variations among these findings may be due to higher poverty levels, different social roles expectation, limited access to medical care, and negative tradition beliefs about epilepsy in LMICs compared to HICs [7]. A number of factors have been found to influence stigma of epilepsy among children and adults including duration of illness, seizure frequency [19], severity and type of illness [22], gender and age of child [13], antiepileptic medication side effects, lower self-efficacy for seizure management, and having epilepsy-related injuries [7, 22]. These factors have been found to vary from one place to another in various studies [23]. This may be due to the different methodological approaches used; sociocultural differences and economic differences in the different geographical locations hence need to harmonize these findings. Additionally, the community’s negative explanatory models of epilepsy (misconceptions) including the belief that epilepsy is untreatable [6], contagious [24], or caused by supernatural powers [7] have been associated with high levels of stigma in persons (children and adults) with epilepsy.

Therefore, this review seeks to document the summative prevalence of enacted and perceived stigma of epilepsy among children and adolescents with epilepsy worldwide and the associated factors. The review will help to inform clinicians about the burden of epilepsy-related stigma in different settings, hence highlighting the need to formulate interventions against the most common influencing factors. These interventions thus can be integrated into the routine care for epilepsy patients so as to improve their management and outcomes.

### Aims

The following are the aims of this study:

1. To determine the prevalence of enacted and perceived stigma of epilepsy among children and adolescents with epilepsy
2. To determine the sociodemographic, psychological, and clinical factors associated with enacted and perceived stigma among children and adolescents with epilepsy

## Methods/design

### Study design

This is a systematic review aiming at determining the prevalence of enacted and perceived stigma of epilepsy and associated factors among children and adolescents globally.

### Review questions

The following are the review questions:

1. What is the prevalence of enacted and perceived stigma among children and adolescents with epilepsy globally?
2. What factors are associated with enacted and perceived stigma among children and adolescents with epilepsy?

### Search strategy

We will conduct electronic searches in PubMed (NLM), EMBASE (Elsevier), PsycINFO (APA), and CINAHL (EBSCOhost) databases from the time of inception up to the time of the date of the last search for the systematic review. We shall search for grey literature from other sources such as students’ theses. We shall also search for further relevant studies via Google Scholar (Google). We shall search for literature by title, abstract, and keywords.

The searches will be done under the following categories (see search strings):

1. Prevalence
2. Epilepsy, convulsions, seizures, and fits
3. Stigma
4. Children and adolescents

We shall search for all studies conducted globally that address the research objectives as shown below:

Terms describing stigma—S1: stigma or prevalence or prevalen* or incidence or inciden* or burden or epidemiology or stereotyping or stereotyp* or social perception or prejudice or label or misconception or discrimination or discriminat*or ignorance or ignoran*

Terms describing child—S2: child or children or adolescent or teen or teenage or youth or child* or adolescen*

Terms describing epilepsy—S3: Epilepsy: epilepsy or epileptic seizures or status epilepticus or convulsion or epilep* or convul*

S4: S1 AND S2 AND S3.

The search process will be presented in a PRISMA flow chart to indicate how many hits were retrieved and inclusion/exclusion justification.

### Study and data management

Studies to be considered for inclusion in this review will be first screened by title and abstract and then by full text at a second screening stage. This will be done by JBF who is a psychiatrist and WA an information scientist supported by AAK, a senior information scientist. The resultant articles will be downloaded and imported into EndNote for screening. Two independent blinded reviewers will extract data about pre-specified variables, that is, AA, an internist with special interest in neurology, and GZR, a senior consultant child and adolescent psychiatrist. In case of any contradiction, it will be resolved by involving a third reviewer (preferably the principal investigator). The data will be extracted using an electronic questionnaire on tablets which will be submitted to an online central server from where it will be exported in an Excel sheet. It will then be availed to the reviewers for analysis.

### Study selection

We shall include observational studies from all parts of the world that report prevalence of either epilepsy-related perceived stigma and/or enacted stigma among children and adolescents aged 2 to 19 years. We shall consider those studies that reported the type of tool or scale they used to measure stigma. The reported tool should be appropriate for the stigma type measured and for the study population. We shall also consider all studies worldwide that reported factors associated with perceived or enacted epilepsy-related stigma among children and adolescents. All relevant articles in all languages from the inception of the databases to the time of the review will be eligible for inclusion in this review.

### Data extraction

Data will be extracted from full text of all screened eligible articles using a pre-piloted extraction form. This will be designed by the investigator and will consist of a baseline study and participant characteristics. This will include sample size, study setting, population duration and aims, year of conducting the study, geographical location (study site), average age, sex distribution and race of participants, type of stigma assessed, stigma scale used, outcomes (mainly prevalence of stigma), and statistical methods used. The form will also include pre-specified factors associated with stigma: gender and age of child, socioeconomic status, duration of illness, seizure frequency, severity and type, side effects of antiepileptic medications, lower self-efficacy for seizure management and having epilepsy-related injuries, antiepileptic drug use, and having misconceptions about cause and contagiousness of epilepsy. All the data will be collected in duplicates (by two independent investigators) to minimize errors [25]. For the data that may be missing from any study, the respective corresponding authors will be contacted accordingly.

### Data synthesis

Analysis will involve both descriptive and quantitative approaches.

A summary narrative will be provided about the studies included in the analysis indicating their characteristics and participant characteristics. A summary table of all included studies will be generated. Heterogeneity will be assessed by visual inspection of the forest plot [26] and chi-squared test [27] and then quantified using the *I*^2^ statistic [28] prior to pooling of studies as per the Cochrane Consumers and Communication Group protocols [29]. In the event that we find statistically significant heterogeneity, then we will report our results as a narrative. Heterogeneity will be further explored by subgroup analysis. The variables for subgroup analysis will include age and residence (study sites) since the prevalence of epilepsy-related stigma is likely to be higher among older children and adolescents from developing countries. In case of homogeneity, then meta-analysis will be done.

### Assessment of bias and quality of evidence

All the studies will be assessed for bias using Munn et al.’s prevalence study critical appraisal tool [30]. This is a 10-item tool with a 4-point Likert score with answers being “no, yes, unclear, or not applicable.” It is easy to use and highly acceptable with face validity. It assesses the appropriateness of the sample and sample size, recruitment procedure, reliability and objectivity of measurements and statistical analysis, and among other parameters. Studies involving assessment of the prevalence of stigma will be assessed for the appropriateness of the stigma tools used. All studies will be checked to ascertain whether they were ethically approved prior to being conducted. The strength of the body of evidence will be assessed using the Grading of Recommendations Assessment, Development and Evaluations (GRADE) approach [31].

### Dissemination plan

A report about the findings will be made available to the Mbarara University of Science and Technology library. A manuscript will also be submitted for review and publication in leading journals of epilepsy or mental health for public access. Findings relevant to clinical practice will be availed to the public, clinicians, health administrators, and national and international health bodies through publication in a peer review journal.

## Discussion

This review will document the prevalence of epilepsy-related stigma and the associated factors among children and adolescents worldwide. It will also determine the different places affected most by this public health challenge and highlight the main factors that need to be addressed to reduce this health challenge. The findings will help in formulating interventions that can be integrated into the routine epilepsy care in order to improve the management and outcomes of epilepsy patients. Since children and adolescents have a longer life span and are the most vulnerable to epilepsy and its social consequences, understanding and addressing this stigma can improve their functionality and productivity in their communities.

## Abbreviations

HIC: High-income counties
MURTI: Mbarara University Research Training Initiative
MUST: Mbarara University of Science and Technology
